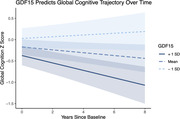# GDF15 as a Prognostic Biomarker of Cognitive Decline in Frontotemporal Dementia

**DOI:** 10.1002/alz.089335

**Published:** 2025-01-09

**Authors:** Coty Chen, Emily W. Paolillo, Rowan Saloner, Anna M. VandeBunte, Claire J. Cadwallader, Shannon Y. Lee, Argentina Lario Lago, Tania F Gendron, Leonard Petrucelli, Julia D Webb, Adam M. Staffaroni, Hilary W. Heuer, Brad Boeve, Leah K. Forsberg, Howard J. Rosen, Julio C. Rojas, Joel H. Kramer, Adam L. Boxer, Kaitlin B. Casaletto, ALLFTD Investigators

**Affiliations:** ^1^ Memory and Aging Center, UCSF Weill Institute for Neurosciences, University of California, San Francisco, San Francisco, CA USA; ^2^ University of Florida, Gainesville, FL USA; ^3^ Memory and Aging Center, UCSF Weill Institute for Neurosciences, University of California San Francisco, San Francisco, CA USA; ^4^ Mayo Clinic, Jacksonville, FL USA; ^5^ University of California San Francisco (UCSF), San Francisco, CA USA; ^6^ Mayo Clinic, Rochester, MN USA; ^7^ Department of Neurology, Mayo Clinic, Rochester, MN USA; ^8^ Memory and Aging Center, Weill Institute for Neurosciences, University of California, San Francisco, San Francisco, CA USA; ^9^ Memory and Aging Center, UCSF Weill Institute for Neurosciences, San Francisco, CA USA

## Abstract

**Background:**

Growth Differentiation Factor 15 (GDF15) is a TGF‐beta superfamily protein upregulated during immune‐mediated stress responses. Peripheral GDF15 is linked to adverse aging across organ systems including the brain. Immune dysregulation is implicated in frontotemporal dementia (FTD) pathogenesis. We examined how plasma GDF15 levels associated with longitudinal cognitive and neurofilament light chain (NfL) trajectories in adults with FTD and controls. Furthermore, we tested the utility of GDF15 as a prognostic biomarker against common inflammatory proteins (CRP and IL6).

**Method:**

231 adults with FTD (24.35% sporadic FTD syndromes; 19.19% MAPT; 19.56% GRN; 22.14% C9orf72 pathogenic carriers) and 40 normal controls from the ALLFTD Consortium completed longitudinal neuropsychological testing and a blood draw with plasma analyzed on SomaScan V4.0 (baseline only: GDF15, CRP, and IL6) and Simoa (longitudinal: NfL). CRP and IL6 were selected as control markers to test the specificity of GDF15. Linear mixed effects models separately examined longitudinal cognition (global, executive functioning, memory, and language z‐scores) and plasma NfL levels as a function of baseline plasma GDF15, time, and their interaction, controlling for baseline age, sex, and education. Parallel models were conducted for CRP and IL6.

**Result:**

In the whole cohort, higher baseline plasma GDF15 levels associated with steeper global cognitive decline (b = ‐3.91, p = 0.0092) and NfL accumulation (b = 3.88, p = 0.00088) over time. When examining cognitive domains, we found GDF15 only associated with worse executive functioning trajectories (b = ‐3.19, p = 0.0021). In contrast, baseline levels of CRP and IL6 did not display statistically significant relationships with cognitive or NfL trajectories. Prognostic effects of GDF15 remained statistically significant when restricting analyses to those with FTD pathogenic variants (b = ‐2.98, p = 0.0054 [global cognition], b = 2.69, p = 0.0098 [NfL]).

**Conclusion:**

Adults with FTD and higher baseline peripheral GDF15 levels exhibit accelerated cognitive decline and neurodegeneration. Our results support GDF15 as a potential prognosticator of adverse clinical outcomes in FTD and highlight the relevance of GDF15‐specific pathways in FTD progression. Plasma GDF15 may provide utility over commonly examined inflammatory proteins and further work to explore its clinical value is indicated.